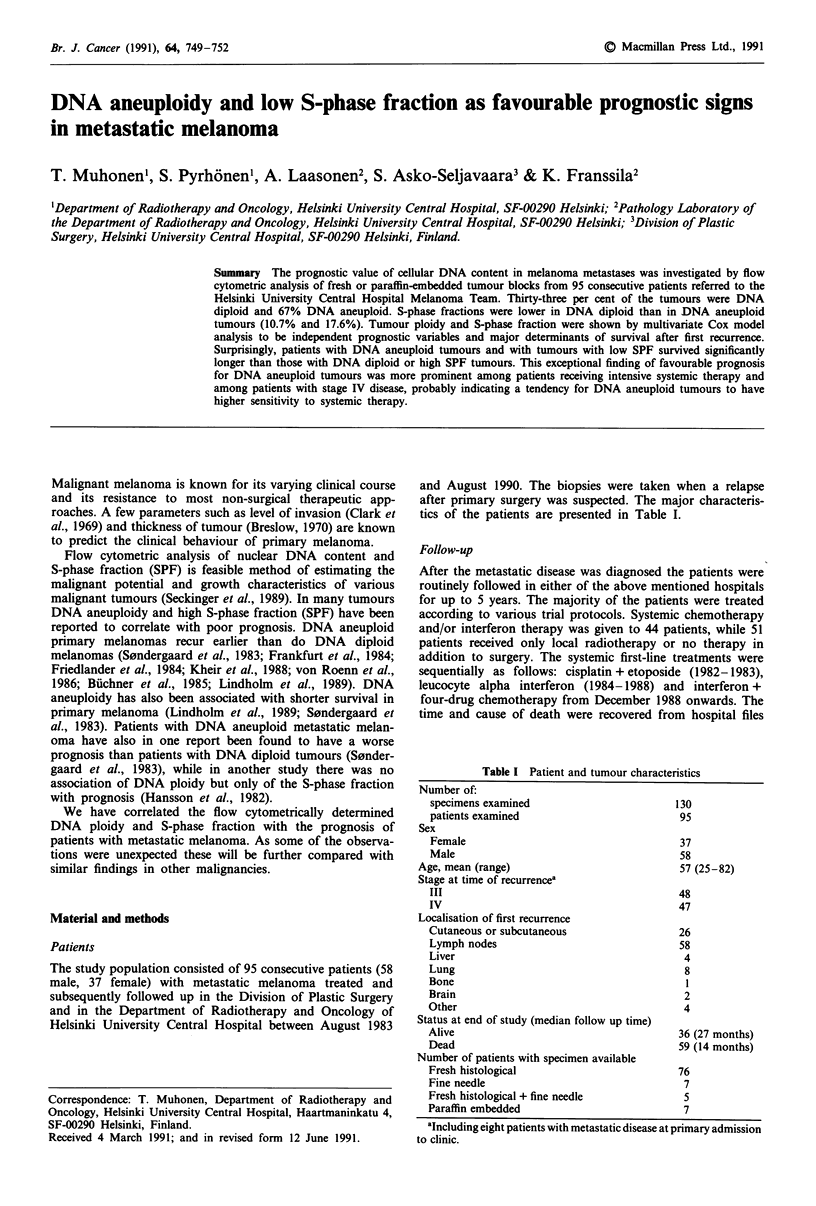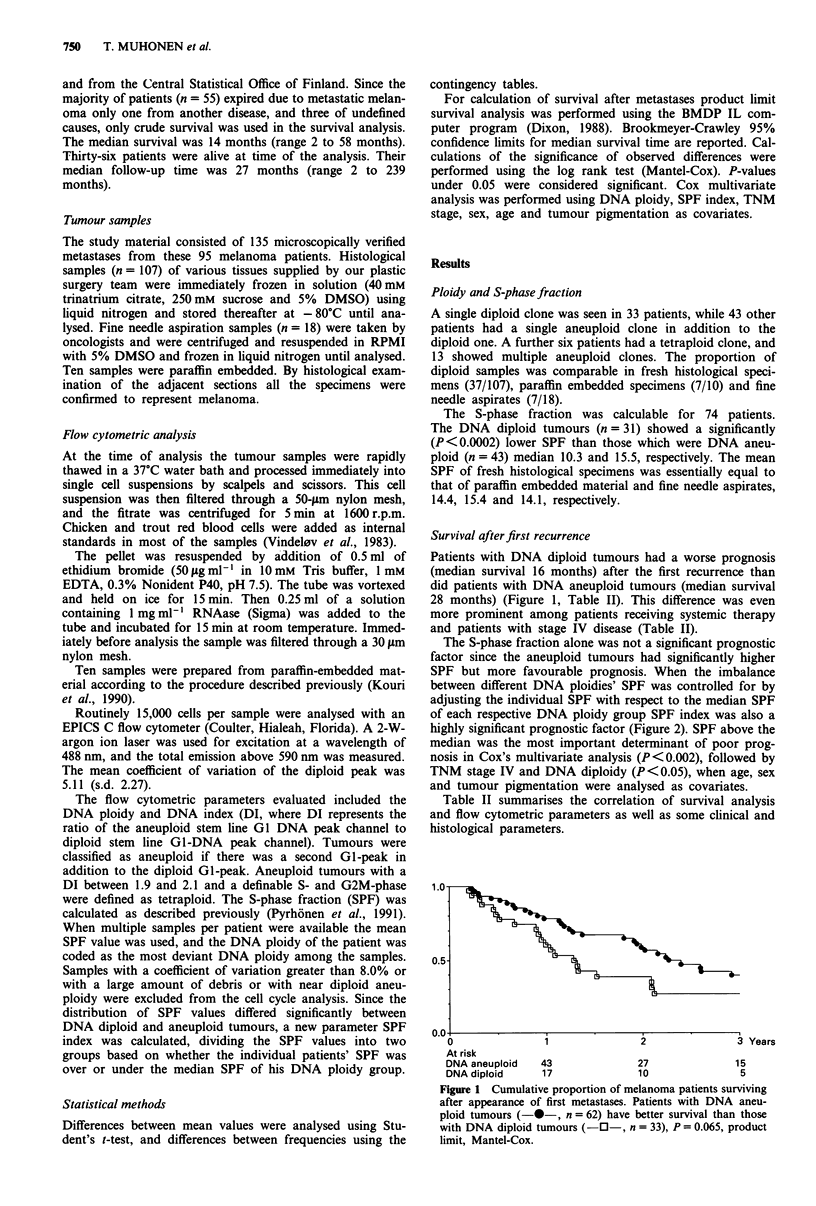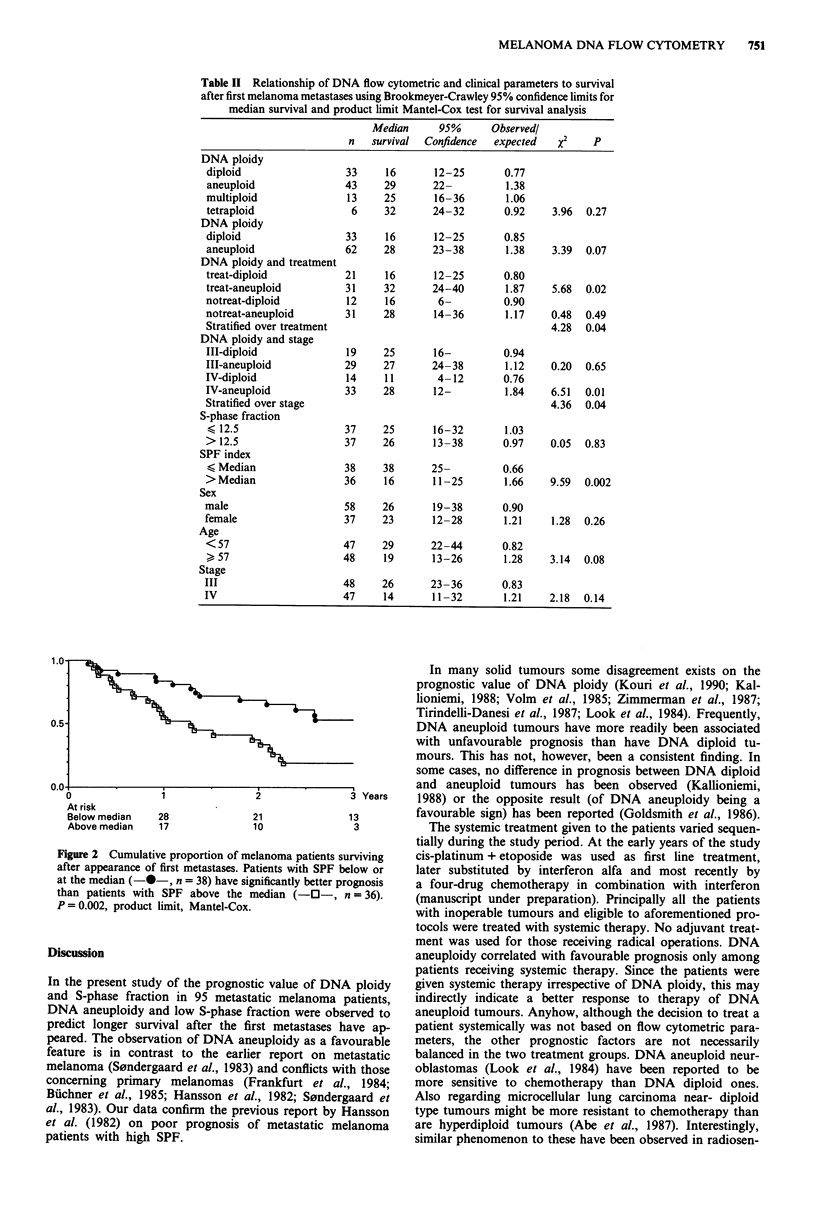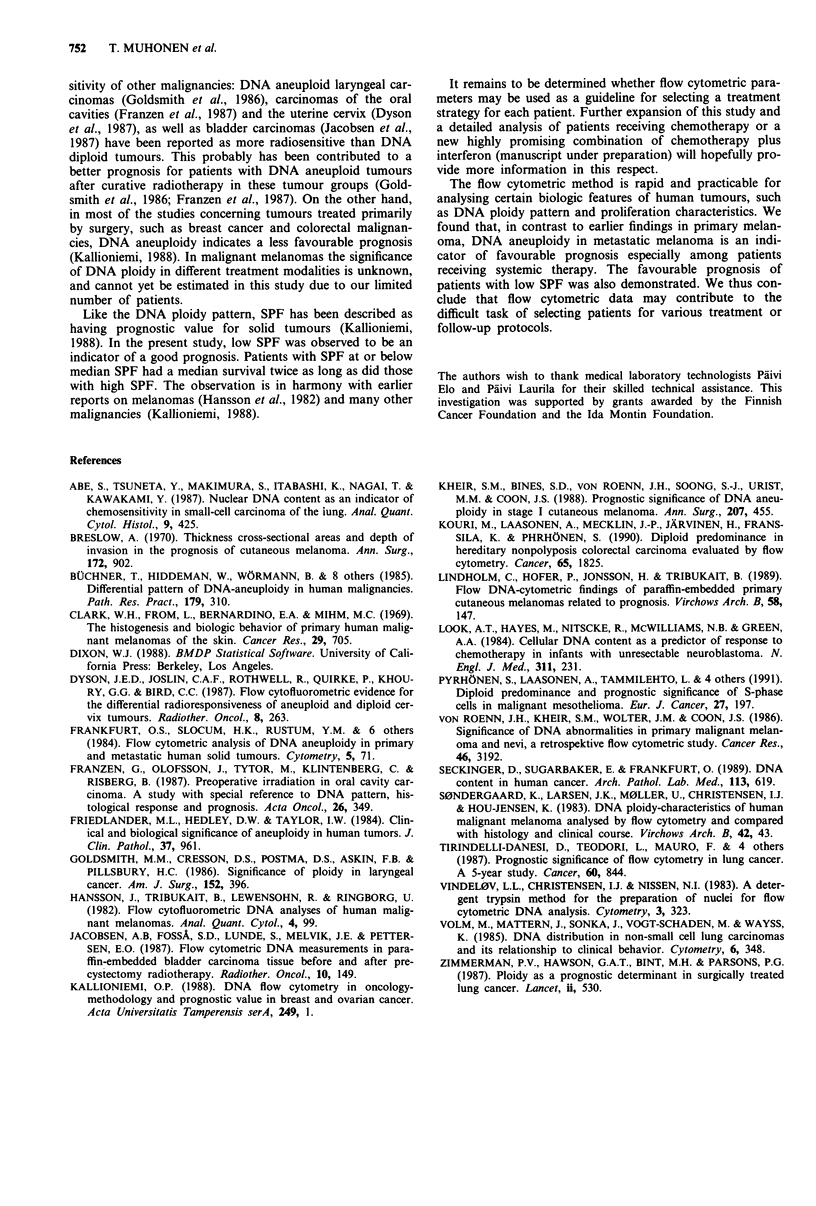# DNA aneuploidy and low S-phase fraction as favourable prognostic signs in metastatic melanoma.

**DOI:** 10.1038/bjc.1991.392

**Published:** 1991-10

**Authors:** T. Muhonen, S. Pyrhönen, A. Laasonen, S. Asko-Seljavaara, K. Franssila

**Affiliations:** Department of Radiotherapy and Oncology, Helsinki University Central Hospital, Finland.

## Abstract

The prognostic value of cellular DNA content in melanoma metastases was investigated by flow cytometric analysis of fresh or paraffin-embedded tumour blocks from 95 consecutive patients referred to the Helsinki University Central Hospital Melanoma Team. Thirty-three per cent of the tumours were DNA diploid and 67% DNA aneuploid. S-phase fractions were lower in DNA diploid than in DNA aneuploid tumours (10.7% and 17.6%). Tumour ploidy and S-phase fraction were shown by multivariate Cox model analysis to be independent prognostic variables and major determinants of survival after first recurrence. Surprisingly, patients with DNA aneuploid tumours and with tumours with low SPF survived significantly longer than those with DNA diploid or high SPF tumours. This exceptional finding of favourable prognosis for DNA aneuploid tumours was more prominent among patients receiving intensive systemic therapy and among patients with stage IV disease, probably indicating a tendency for DNA aneuploid tumours to have higher sensitivity to systemic therapy.


Br. J. Cancer (1991), 64, 749-752 © Macmillan Press Ltd., 1991

### DNA aneuploidy and low S-phase fraction as favourable prognostic signs in metastatic melanoma

T. Muhonen[1], S. Pyrhönen[1], A. Laasonen[2], S. Asko-Seljavaara[3] & K. Franssila[2]

[1]*Department of Radiotherapy and Oncology, Helsinki University Central Hospital, SF-00290 Helsinki;* [2]*Pathology Laboratory of the Department of Radiotherapy and Oncology, Helsinki University Central Hospital, SF-00290 Helsinki;* [3]*Division of Plastic Surgery, Helsinki University Central Hospital, SF-00290 Helsinki, Finland.*

**Summary** The prognostic value of cellular DNA content in melanoma metastases was investigated by flow cytometric analysis of fresh or paraffin-embedded tumour blocks from 95 consecutive patients referred to the Helsinki University Central Hospital Melanoma Team. Thirty-three per cent of the tumours were DNA diploid and 67% DNA aneuploid. S-phase fractions were lower in DNA diploid than in DNA aneuploid tumours (10.7% and 17.6%). Tumour ploidy and S-phase fraction were shown by multivariate Cox model analysis to be independent prognostic variables and major determinants of survival after first recurrence. Surprisingly, patients with DNA aneuploid tumours and with tumours with low SPF survived significantly longer than those with DNA diploid or high SPF tumours. This exceptional finding of favourable prognosis for DNA aneuploid tumours was more prominent among patients receiving intensive systemic therapy and among patients with stage IV disease, probably indicating a tendency for DNA aneuploid tumours to have higher sensitivity to systemic therapy.

Malignant melanoma is known for its varying clinical course and its resistance to most non-surgical therapeutic approaches. A few parameters such as level of invasion (Clark *et al.*, 1969) and thickness of tumour (Breslow, 1970) are known to predict the clinical behaviour of primary melanoma.

Flow cytometric analysis of nuclear DNA content and S-phase fraction (SPF) is feasible method of estimating the malignant potential and growth characteristics of various malignant tumours (Seckinger *et al.*, 1989). In many tumours DNA aneuploidy and high S-phase fraction (SPF) have been reported to correlate with poor prognosis. DNA aneuploid primary melanomas recur earlier than do DNA diploid melanomas (Søndergaard *et al.*, 1983; Frankfurt *et al.*, 1984; Friedlander *et al.*, 1984; Kheir *et al.*, 1988; von Roenn *et al.*, 1986; Büchner *et al.*, 1985; Lindholm *et al.*, 1989). DNA aneuploidy has also been associated with shorter survival in primary melanoma (Lindholm *et al.*, 1989; Søndergaard *et al.*, 1983). Patients with DNA aneuploid metastatic melanoma have also in one report been found to have a worse prognosis than patients with DNA diploid tumours (Søndergaard *et al.*, 1983), while in another study there was no association of DNA ploidy but only of the S-phase fraction with prognosis (Hansson *et al.*, 1982).

We have correlated the flow cytometrically determined DNA ploidy and S-phase fraction with the prognosis of patients with metastatic melanoma. As some of the observations were unexpected these will be further compared with similar findings in other malignancies.

### Material and methods

### *Patients*

The study population consisted of 95 consecutive patients (58 male, 37 female) with metastatic melanoma treated and subsequently followed up in the Division of Plastic Surgery and in the Department of Radiotherapy and Oncology of Helsinki University Central Hospital between August 1983 and August 1990. The biopsies were taken when a relapse after primary surgery was suspected. The major characteristics of the patients are presented in Table I.

### *Follow-up*

After the metastatic disease was diagnosed the patients were routinely followed in either of the above mentioned hospitals for up to 5 years. The majority of the patients were treated according to various trial protocols. Systemic chemotherapy and/or interferon therapy was given to 44 patients, while 51 patients received only local radiotherapy or no therapy in addition to surgery. The systemic first-line treatments were sequentially as follows: cisplatin + etoposide (1982-1983), leucocyte alpha interferon (1984-1988) and interferon + four-drug chemotherapy from December 1988 onwards. The time and cause of death were recovered from hospital files

**Table I** Patient and tumour characteristics

| | |
|---|---|
| Number of: | |
| specimens examined | 130 |
| patients examined | 95 |
| Sex | |
| Female | 37 |
| Male | 58 |
| Age, mean (range) | 57 (25-82) |
| Stage at time of recurrence[a] | |
| III | 48 |
| IV | 47 |
| Localisation of first recurrence | |
| Cutaneous or subcutaneous | 26 |
| Lymph nodes | 58 |
| Liver | 4 |
| Lung | 8 |
| Bone | 1 |
| Brain | 2 |
| Other | 4 |
| Status at end of study (median follow up time) | |
| Alive | 36 (27 months) |
| Dead | 59 (14 months) |
| Number of patients with specimen available | |
| Fresh histological | 76 |
| Fine needle | 7 |
| Fresh histological + fine needle | 5 |
| Paraffin embedded | 7 |

[a]Including eight patients with metastatic disease at primary admission to clinic.

Correspondence: T. Muhonen, Department of Radiotherapy and Oncology, Helsinki University Central Hospital, Haartmaninkatu 4, SF-00290 Helsinki, Finland.
Received 4 March 1991; and in revised form 12 June 1991.

and from the Central Statistical Office of Finland. Since the majority of patients ($n = 55$) expired due to metastatic melanoma only one from another disease, and three of undefined causes, only crude survival was used in the survival analysis. The median survival was 14 months (range 2 to 58 months). Thirty-six patients were alive at time of the analysis. Their median follow-up time was 27 months (range 2 to 239 months).

*Tumour samples*

The study material consisted of 135 microscopically verified metastases from these 95 melanoma patients. Histological samples ($n = 107$) of various tissues supplied by our plastic surgery team were immediately frozen in solution (40 mM trinatrium citrate, 250 mM sucrose and 5% DMSO) using liquid nitrogen and stored thereafter at −80°C until analysed. Fine needle aspiration samples ($n = 18$) were taken by oncologists and were centrifuged and resuspended in RPMI with 5% DMSO and frozen in liquid nitrogen until analysed. Ten samples were paraffin embedded. By histological examination of the adjacent sections all the specimens were confirmed to represent melanoma.

*Flow cytometric analysis*

At the time of analysis the tumour samples were rapidly thawed in a 37°C water bath and processed immediately into single cell suspensions by scalpels and scissors. This cell suspension was then filtered through a 50-μm nylon mesh, and the fitrate was centrifuged for 5 min at 1600 r.p.m. Chicken and trout red blood cells were added as internal standards in most of the samples (Vindeløv *et al.*, 1983).

The pellet was resuspended by addition of 0.5 ml of ethidium bromide (50 μg ml$^{-1}$ in 10 mM Tris buffer, 1 mM EDTA, 0.3% Nonident P40, pH 7.5). The tube was vortexed and held on ice for 15 min. Then 0.25 ml of a solution containing 1 mg ml$^{-1}$ RNAase (Sigma) was added to the tube and incubated for 15 min at room temperature. Immediately before analysis the sample was filtered through a 30 μm nylon mesh.

Ten samples were prepared from paraffin-embedded material according to the procedure described previously (Kouri *et al.*, 1990).

Routinely 15,000 cells per sample were analysed with an EPICS C flow cytometer (Coulter, Hialeah, Florida). A 2-W-argon ion laser was used for excitation at a wavelength of 488 nm, and the total emission above 590 nm was measured. The mean coefficient of variation of the diploid peak was 5.11 (s.d. 2.27).

The flow cytometric parameters evaluated included the DNA ploidy and DNA index (DI, where DI represents the ratio of the aneuploid stem line G1 DNA peak channel to diploid stem line G1-DNA peak channel). Tumours were classified as aneuploid if there was a second G1-peak in addition to the diploid G1-peak. Aneuploid tumours with a DI between 1.9 and 2.1 and a definable S- and G2M-phase were defined as tetraploid. The S-phase fraction (SPF) was calculated as described previously (Pyrhönen *et al.*, 1991). When multiple samples per patient were available the mean SPF value was used, and the DNA ploidy of the patient was coded as the most deviant DNA ploidy among the samples. Samples with a coefficient of variation greater than 8.0% or with a large amount of debris or with near diploid aneuploidy were excluded from the cell cycle analysis. Since the distribution of SPF values differed significantly between DNA diploid and aneuploid tumours, a new parameter SPF index was calculated, dividing the SPF values into two groups based on whether the individual patients' SPF was over or under the median SPF of his DNA ploidy group.

*Statistical methods*

Differences between mean values were analysed using Student's *t*-test, and differences between frequencies using the contingency tables.

For calculation of survival after metastases product limit survival analysis was performed using the BMDP IL computer program (Dixon, 1988). Brookmeyer-Crawley 95% confidence limits for median survival time are reported. Calculations of the significance of observed differences were performed using the log rank test (Mantel-Cox). *P*-values under 0.05 were considered significant. Cox multivariate analysis was performed using DNA ploidy, SPF index, TNM stage, sex, age and tumour pigmentation as covariates.

### Results

*Ploidy and S-phase fraction*

A single diploid clone was seen in 33 patients, while 43 other patients had a single aneuploid clone in addition to the diploid one. A further six patients had a tetraploid clone, and 13 showed multiple aneuploid clones. The proportion of diploid samples was comparable in fresh histological specimens (37/107), paraffin embedded specimens (7/10) and fine needle aspirates (7/18).

The S-phase fraction was calculable for 74 patients. The DNA diploid tumours ($n = 31$) showed a significantly ($P < 0.0002$) lower SPF than those which were DNA aneuploid ($n = 43$) median 10.3 and 15.5, respectively. The mean SPF of fresh histological specimens was essentially equal to that of paraffin embedded material and fine needle aspirates, 14.4, 15.4 and 14.1, respectively.

*Survival after first recurrence*

Patients with DNA diploid tumours had a worse prognosis (median survival 16 months) after the first recurrence than did patients with DNA aneuploid tumours (median survival 28 months) (Figure 1, Table II). This difference was even more prominent among patients receiving systemic therapy and patients with stage IV disease (Table II).

The S-phase fraction alone was not a significant prognostic factor since the aneuploid tumours had significantly higher SPF but more favourable prognosis. When the imbalance between different DNA ploidies' SPF was controlled for by adjusting the individual SPF with respect to the median SPF of each respective DNA ploidy group SPF index was also a highly significant prognostic factor (Figure 2). SPF above the median was the most important determinant of poor prognosis in Cox's multivariate analysis ($P < 0.002$), followed by TNM stage IV and DNA diploidy ($P < 0.05$), when age, sex and tumour pigmentation were analysed as covariates.

Table II summarises the correlation of survival analysis and flow cytometric parameters as well as some clinical and histological parameters.

**Figure 1** Cumulative proportion of melanoma patients surviving after appearance of first metastases. Patients with DNA aneuploid tumours (—●—, $n = 62$) have better survival than those with DNA diploid tumours (—□—, $n = 33$), $P = 0.065$, product limit, Mantel-Cox.

**Table II** Relationship of DNA flow cytometric and clinical parameters to survival after first melanoma metastases using Brookmeyer-Crawley 95% confidence limits for median survival and product limit Mantel-Cox test for survival analysis

| | *n* | *Median survival* | *95% Confidence* | *Observed/ expected* | $\chi^2$ | *P* |
|---|---|---|---|---|---|---|
| DNA ploidy | | | | | | |
| diploid | 33 | 16 | 12-25 | 0.77 | | |
| aneuploid | 43 | 29 | 22- | 1.38 | | |
| multiploid | 13 | 25 | 16-36 | 1.06 | | |
| tetraploid | 6 | 32 | 24-32 | 0.92 | 3.96 | 0.27 |
| DNA ploidy | | | | | | |
| diploid | 33 | 16 | 12-25 | 0.85 | | |
| aneuploid | 62 | 28 | 23-38 | 1.38 | 3.39 | 0.07 |
| DNA ploidy and treatment | | | | | | |
| treat-diploid | 21 | 16 | 12-25 | 0.80 | | |
| treat-aneuploid | 31 | 32 | 24-40 | 1.87 | 5.68 | 0.02 |
| notreat-diploid | 12 | 16 | 6- | 0.90 | | |
| notreat-aneuploid | 31 | 28 | 14-36 | 1.17 | 0.48 | 0.49 |
| Stratified over treatment | | | | | 4.28 | 0.04 |
| DNA ploidy and stage | | | | | | |
| III-diploid | 19 | 25 | 16- | 0.94 | | |
| III-aneuploid | 29 | 27 | 24-38 | 1.12 | 0.20 | 0.65 |
| IV-diploid | 14 | 11 | 4-12 | 0.76 | | |
| IV-aneuploid | 33 | 28 | 12- | 1.84 | 6.51 | 0.01 |
| Stratified over stage | | | | | 4.36 | 0.04 |
| S-phase fraction | | | | | | |
| $\leqslant$ 12.5 | 37 | 25 | 16-32 | 1.03 | | |
| > 12.5 | 37 | 26 | 13-38 | 0.97 | 0.05 | 0.83 |
| SPF index | | | | | | |
| $\leqslant$ Median | 38 | 38 | 25- | 0.66 | | |
| > Median | 36 | 16 | 11-25 | 1.66 | 9.59 | 0.002 |
| Sex | | | | | | |
| male | 58 | 26 | 19-38 | 0.90 | | |
| female | 37 | 23 | 12-28 | 1.21 | 1.28 | 0.26 |
| Age | | | | | | |
| < 57 | 47 | 29 | 22-44 | 0.82 | | |
| $\geqslant$ 57 | 48 | 19 | 13-26 | 1.28 | 3.14 | 0.08 |
| Stage | | | | | | |
| III | 48 | 26 | 23-36 | 0.83 | | |
| IV | 47 | 14 | 11-32 | 1.21 | 2.18 | 0.14 |

**Figure 2** Cumulative proportion of melanoma patients surviving after appearance of first metastases. Patients with SPF below or at the median (—●—, $n = 38$) have significantly better prognosis than patients with SPF above the median (—□—, $n = 36$). $P = 0.002$, product limit, Mantel-Cox.

### Discussion

In the present study of the prognostic value of DNA ploidy and S-phase fraction in 95 metastatic melanoma patients, DNA aneuploidy and low S-phase fraction were observed to predict longer survival after the first metastases have appeared. The observation of DNA aneuploidy as a favourable feature is in contrast to the earlier report on metastatic melanoma (Søndergaard *et al.*, 1983) and conflicts with those concerning primary melanomas (Frankfurt *et al.*, 1984; Büchner *et al.*, 1985; Hansson *et al.*, 1982; Søndergaard *et al.*, 1983). Our data confirm the previous report by Hansson *et al.* (1982) on poor prognosis of metastatic melanoma patients with high SPF.

In many solid tumours some disagreement exists on the prognostic value of DNA ploidy (Kouri *et al.*, 1990; Kallioniemi, 1988; Volm *et al.*, 1985; Zimmerman *et al.*, 1987; Tirindelli-Danesi *et al.*, 1987; Look *et al.*, 1984). Frequently, DNA aneuploid tumours have more readily been associated with unfavourable prognosis than have DNA diploid tumours. This has not, however, been a consistent finding. In some cases, no difference in prognosis between DNA diploid and aneuploid tumours has been observed (Kallioniemi, 1988) or the opposite result (of DNA aneuploidy being a favourable sign) has been reported (Goldsmith *et al.*, 1986).

The systemic treatment given to the patients varied sequentially during the study period. At the early years of the study cis-platinum + etoposide was used as first line treatment, later substituted by interferon alfa and most recently by a four-drug chemotherapy in combination with interferon (manuscript under preparation). Principally all the patients with inoperable tumours and eligible to aforementioned protocols were treated with systemic therapy. No adjuvant treatment was used for those receiving radical operations. DNA aneuploidy correlated with favourable prognosis only among patients receiving systemic therapy. Since the patients were given systemic therapy irrespective of DNA ploidy, this may indirectly indicate a better response to therapy of DNA aneuploid tumours. Anyhow, although the decision to treat a patient systemically was not based on flow cytometric parameters, the other prognostic factors are not necessarily balanced in the two treatment groups. DNA aneuploid neuroblastomas (Look *et al.*, 1984) have been reported to be more sensitive to chemotherapy than DNA diploid ones. Also regarding microcellular lung carcinoma near- diploid type tumours might be more resistant to chemotherapy than are hyperdiploid tumours (Abe *et al.*, 1987). Interestingly, similar phenomenon to these have been observed in radiosensitivity of other malignancies: DNA aneuploid laryngeal carcinomas (Goldsmith et al., 1986), carcinomas of the oral cavities (Franzen et al., 1987) and the uterine cervix (Dyson et al., 1987), as well as bladder carcinomas (Jacobsen et al., 1987) have been reported as more radiosensitive than DNA diploid tumours. This probably has been contributed to a better prognosis for patients with DNA aneuploid tumours after curative radiotherapy in these tumour groups (Goldsmith et al., 1986; Franzen et al., 1987). On the other hand, in most of the studies concerning tumours treated primarily by surgery, such as breast cancer and colorectal malignancies, DNA aneuploidy indicates a less favourable prognosis (Kallioniemi, 1988). In malignant melanomas the significance of DNA ploidy in different treatment modalities is unknown, and cannot yet be estimated in this study due to our limited number of patients.

Like the DNA ploidy pattern, SPF has been described as having prognostic value for solid tumours (Kallioniemi, 1988). In the present study, low SPF was observed to be an indicator of a good prognosis. Patients with SPF at or below median SPF had a median survival twice as long as did those with high SPF. The observation is in harmony with earlier reports on melanomas (Hansson et al., 1982) and many other malignancies (Kallioniemi, 1988).

It remains to be determined whether flow cytometric parameters may be used as a guideline for selecting a treatment strategy for each patient. Further expansion of this study and a detailed analysis of patients receiving chemotherapy or a new highly promising combination of chemotherapy plus interferon (manuscript under preparation) will hopefully provide more information in this respect.

The flow cytometric method is rapid and practicable for analysing certain biologic features of human tumours, such as DNA ploidy pattern and proliferation characteristics. We found that, in contrast to earlier findings in primary melanoma, DNA aneuploidy in metastatic melanoma is an indicator of favourable prognosis especially among patients receiving systemic therapy. The favourable prognosis of patients with low SPF was also demonstrated. We thus conclude that flow cytometric data may contribute to the difficult task of selecting patients for various treatment or follow-up protocols.

The authors wish to thank medical laboratory technologists Päivi Elo and Päivi Laurila for their skilled technical assistance. This investigation was supported by grants awarded by the Finnish Cancer Foundation and the Ida Montin Foundation.

### References

ABE, S., TSUNETA, Y., MAKIMURA, S., ITABASHI, K., NAGAI, T. & KAWAKAMI, Y. (1987). Nuclear DNA content as an indicator of chemosensitivity in small-cell carcinoma of the lung. *Anal. Quant. Cytol. Histol.*, **9**, 425.

BRESLOW, A. (1970). Thickness cross-sectional areas and depth of invasion in the prognosis of cutaneous melanoma. *Ann. Surg.*, **172**, 902.

BÜCHNER, T., HIDDEMAN, W., WÖRMANN, B. & 8 others (1985). Differential pattern of DNA-aneuploidy in human malignancies. *Path. Res. Pract.*, **179**, 310.

CLARK, W.H., FROM, L., BERNARDINO, E.A. & MIHM, M.C. (1969). The histogenesis and biologic behavior of primary human malignant melanomas of the skin. *Cancer Res.*, **29**, 705.

DIXON, W.J. (1988). *BMDP Statistical Software*. University of California Press: Berkeley, Los Angeles.

DYSON, J.E.D., JOSLIN, C.A.F., ROTHWELL, R., QUIRKE, P., KHOURY, G.G. & BIRD, C.C. (1987). Flow cytofluorometric evidence for the differential radioresponsiveness of aneuploid and diploid cervix tumours. *Radiother. Oncol.*, **8**, 263.

FRANKFURT, O.S., SLOCUM, H.K., RUSTUM, Y.M. & 6 others (1984). Flow cytometric analysis of DNA aneuploidy in primary and metastatic human solid tumours. *Cytometry*, **5**, 71.

FRANZEN, G., OLOFSSON, J., TYTOR, M., KLINTENBERG, C. & RISBERG, B. (1987). Preoperative irradiation in oral cavity carcinoma. A study with special reference to DNA pattern, histological response and prognosis. *Acta Oncol.*, **26**, 349.

FRIEDLANDER, M.L., HEDLEY, D.W. & TAYLOR, I.W. (1984). Clinical and biological significance of aneuploidy in human tumors. *J. Clin. Pathol.*, **37**, 961.

GOLDSMITH, M.M., CRESSON, D.S., POSTMA, D.S., ASKIN, F.B. & PILLSBURY, H.C. (1986). Significance of ploidy in laryngeal cancer. *Am. J. Surg.*, **152**, 396.

HANSSON, J., TRIBUKAIT, B., LEWENSOHN, R. & RINGBORG, U. (1982). Flow cytofluorometric DNA analyses of human malignant melanomas. *Anal. Quant. Cytol.*, **4**, 99.

JACOBSEN, A.B, FOSSÅ, S.D., LUNDE, S., MELVIK, J.E. & PETTERSEN, E.O. (1987). Flow cytometric DNA measurements in paraffin-embedded bladder carcinoma tissue before and after precystectomy radiotherapy. *Radiother. Oncol.*, **10**, 149.

KALLIONIEMI, O.P. (1988). DNA flow cytometry in oncology-methodology and prognostic value in breast and ovarian cancer. *Acta Universitatis Tamperensis serA*, **249**, 1.

KHEIR, S.M., BINES, S.D., VON ROENN, J.H., SOONG, S.-J., URIST, M.M. & COON, J.S. (1988). Prognostic significance of DNA aneuploidy in stage I cutaneous melanoma. *Ann. Surg.*, **207**, 455.

KOURI, M., LAASONEN, A., MECKLIN, J.-P., JÄRVINEN, H., FRANSSILA, K. & PHRHÖNEN, S. (1990). Diploid predominance in hereditary nonpolyposis colorectal carcinoma evaluated by flow cytometry. *Cancer*, **65**, 1825.

LINDHOLM, C., HOFER, P., JONSSON, H. & TRIBUKAIT, B. (1989). Flow DNA-cytometric findings of paraffin-embedded primary cutaneous melanomas related to prognosis. *Virchows Arch. B*, **58**, 147.

LOOK, A.T., HAYES, M., NITSCKE, R., MCWILLIAMS, N.B. & GREEN, A.A. (1984). Cellular DNA content as a predictor of response to chemotherapy in infants with unresectable neuroblastoma. *N. Engl. J. Med.*, **311**, 231.

PYRHÖNEN, S., LAASONEN, A., TAMMILEHTO, L. & 4 others (1991). Diploid predominance and prognostic significance of S-phase cells in malignant mesothelioma. *Eur. J. Cancer*, **27**, 197.

VON ROENN, J.H., KHEIR, S.M., WOLTER, J.M. & COON, J.S. (1986). Significance of DNA abnormalities in primary malignant melanoma and nevi, a retrospektive flow cytometric study. *Cancer Res.*, **46**, 3192.

SECKINGER, D., SUGARBAKER, E. & FRANKFURT, O. (1989). DNA content in human cancer. *Arch. Pathol. Lab. Med.*, **113**, 619.

SØNDERGAARD, K., LARSEN, J.K., MØLLER, U., CHRISTENSEN, I.J. & HOU-JENSEN, K. (1983). DNA ploidy-characteristics of human malignant melanoma analysed by flow cytometry and compared with histology and clinical course. *Virchows Arch. B*, **42**, 43.

TIRINDELLI-DANESI, D., TEODORI, L., MAURO, F. & 4 others (1987). Prognostic significance of flow cytometry in lung cancer. A 5-year study. *Cancer*, **60**, 844.

VINDELØV, L.L., CHRISTENSEN, I.J. & NISSEN, N.I. (1983). A detergent trypsin method for the preparation of nuclei for flow cytometric DNA analysis. *Cytometry*, **3**, 323.

VOLM, M., MATTERN, J., SONKA, J., VOGT-SCHADEN, M. & WAYSS, K. (1985). DNA distribution in non-small cell lung carcinomas and its relationship to clinical behavior. *Cytometry*, **6**, 348.

ZIMMERMAN, P.V., HAWSON, G.A.T., BINT, M.H. & PARSONS, P.G. (1987). Ploidy as a prognostic determinant in surgically treated lung cancer. *Lancet*, **ii**, 530.